# Optimized methyl donor and reduced precursor degradation pathway for seleno-methylselenocysteine production in *Bacillus subtilis*

**DOI:** 10.1186/s12934-023-02203-1

**Published:** 2023-10-19

**Authors:** Xian Yin, Meiyi Zhao, Yu Zhou, Hulin Yang, Yonghong Liao, Fenghuan Wang

**Affiliations:** 1 Key Laboratory of Geriatric Nutrition and Health (Ministry of Education), Beijing Technology and Business University, Fucheng RD 11, Beijing, 100048 China; 2 China Food Flavor and Nutrition Health Innovation Center, Beijing Technology and Business University, Fucheng RD 11, Beijing, 100048 China; 3 School of Light Industry, Beijing Technology and Business University, Fucheng RD 11, Beijing, 100048 China

**Keywords:** Seleno-methylselenocysteine, Methylmethionine, Selenocysteine, Serine, *Bacillus subtilis*

## Abstract

**Background:**

Seleno-methylselenocysteine (SeMCys) is an effective component of selenium supplementation with anti-carcinogenic potential that can ameliorate neuropathology and cognitive deficits. In a previous study, a SeMCys producing strain of *Bacillus subtilis* GBACB was generated by releasing feedback inhibition by overexpression of cysteine-insensitive serine *O*-acetyltransferase, enhancing the synthesis of *S*-adenosylmethionine as methyl donor by overexpression of *S*-adenosylmethionine synthetase, and expressing heterologous selenocysteine methyltransferase. In this study, we aimed to improve GBACB SeMCys production by synthesizing methylmethionine as a donor to methylate selenocysteine and by inhibiting the precursor degradation pathway.

**Results:**

First, the performance of three methionine *S*-methyltransferases that provide methylmethionine as a methyl donor for SeMCys production was determined. Integration of the *NmMmt* gene into GBACB improved SeMCys production from 20.7 to 687.4 μg/L. Next, the major routes for the degradation of selenocysteine, which is the precursor of SeMCys, were revealed by comparing selenocysteine hyper-accumulating and non-producing strains at the transcriptional level. The *iscSB* knockout strain doubled SeMCys production. Moreover, deleting *sdaA*, which is responsible for the degradation of serine as a precursor of selenocysteine, enhanced SeMCys production to 4120.3 μg/L. Finally, the culture conditions in the flasks were optimized. The strain was tolerant to higher selenite content in the liquid medium and the titer of SeMCys reached 7.5 mg/L.

**Conclusions:**

The significance of methylmethionine as a methyl donor for SeMCys production in *B. subtilis* is reported, and enhanced precursor supply facilitates SeMCys synthesis. The results represent the highest SeMCys production to date and provide insight into Se metabolism.

**Supplementary Information:**

The online version contains supplementary material available at 10.1186/s12934-023-02203-1.

## Background

Selenium (Se) is a vital trace element in diverse organisms that can be inserted into proteins and nucleic acids via selenocysteine (SeCys) and 2-selenouridine. There are diverse selenium-containing small molecules, including free selenoamino acids, selenosugars, Se-containing peptides [1] and selenoneine [2]. Se-methylselenocysteine (SeMCys), a direct precursor of methylselenol, can be used as a dietary Se supplement and applied in combination with chemotherapy to reduce tumor growth and metastatic ability [3]. It can be applied as part of novel therapeutic approaches for the treatment of visceral organs [4] and as an anti-oxidant to protect normal tissues and organs from chemotherapy-induced systemic toxicity [5].

**Fig. 1:**
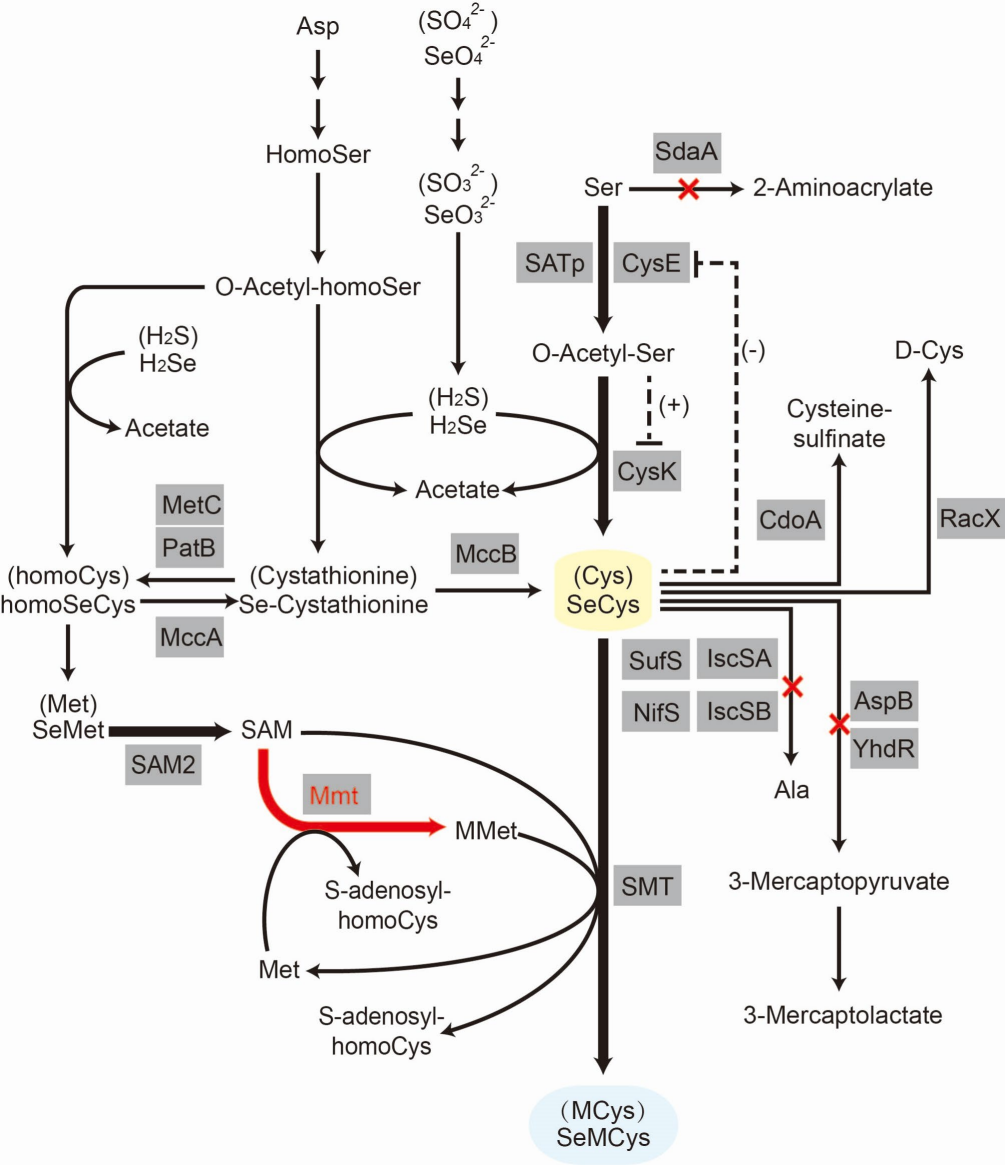
Pathway engineering of *B. subtilis* for SeMCys biosynthesis. Ala, alanine; Asp, Aspartate; Cys, cysteine; D-Cys, D-cysteine; HomoCys, Homocysteine; HomoSeCys, selenohomocysteine; HomoSer, Homoserine; MCys, methylcysteine; Met, methionine; MMet, methylmethionine; O-Acetyl-homoSer, *O*-Acetyl-homoserine; O-Acetyl-Ser, *O*-acetylserine; *S*-adenosyl-homoCys, *S*-adenosyl-homocysteine; SAM, *S*-adenosylmethioine; SeCys, selenocysteine; *Se*-Cystathionine, selenocystathionine; SeMCys, seleno-methylselenocysteine; SeMet, selenomethionine; Ser, serine. Black arrow, enhanced steps in the previous study; red arrow, enhanced step in the present study; dash line, feedback repression or feedback inhibition; plus sign, positive control; minus sign, negative control; red cross, pathway knocked out in this study

Biosynthesis of SeMCys occurs naturally in broccoli and species of *Astragalus*, which are Se-hyperaccumulating plants [6, 7]. Selenocysteine methyltransferase (SMT) is a key enzyme responsible for methylating SeCys to synthesize SeMCys [8]. The expression of plant derived SMT in *Saccharomyces cerevisiae* overproducing *S*-adenosylmethioine (SAM) as a methyl donor produces SeMCys at 1.140 μg/g dry cell weight (DCW) [9]. Based on the over-expression of SMT and improved SAM production by expressing *S*-adenosylmethionine synthetase (SAM2) from *S. cerevisiae*, our previous work enhanced intracellular SeCys levels to optimize the production of SeMCys in *Bacillus subtilis* (Fig. 1), and the heterologous gene integration strain produced SeMCys at 18 μg/L [10].

Although SAM is the major methyl donor in many transmethylation reactions, it has been demonstrated that specific SMT activity of *Astragalus bisulcatus* is four times higher when using *S*-methylmethionine (MMet) as a methyl donor substrate than when using SAM [11]. MMet has been detected in angiosperms and heterotrophic marine bacteria. It plays a major role in sulfur transport in flowering plants, and is formed from methionine (Met) via the action of Met *S*-methyltransferases (MMTs) [12]. In marine bacteria, MMet is used as an intermediate in the methylation pathway to produce dimethylsulfoniopropionate from Met [13], and the corresponding enzyme Mmt has been predicted via “omics” [14]. According to protein sequence homology analysis, the Mmt family can be divided into three groups. In the first group, plant derived MMT contains two parts: the N-terminal domain for methylating Met and the C-terminal aminotransferase domain. The other two groups consist of marine bacteria-originated Mmts, which are much smaller with only one domain that is approximately 30% identical to the N-terminal domain of plant MMT [15]. RiMmt and NmMmt belong to the second group. The crystal structure of the purified RiMmt shows three molecules arranged as a trimer in the asymmetric unit. RiMmt exhibits *K*_m_ values of 6.2 mM for SAM and 15.3 mM for Met at pH 8.0 and 30 °C with a *k*_cat_ value of 1.1 min^− 1^ [15]. NmMmt exhibits *K*_m_ values of 2.0 mM for Met and 1.0 mM for SAM [16]. In the third group, CpMmt catalyzes SAM to form *S*-adenosyl-homocysteine, indicating MMet synthesis [15]. However, the effect of Mmt on SeMCys production in *B. subtilis* under complex intracellular metabolic circumstance needs to be verified.

To improve SeMCys production, a continuous supply of high levels of SeCys, the precursor of SeMCys, is crucial. The formation of SeCys depends on the sulfur metabolic pathway and the corresponding metabolite is cysteine (Cys), which is crucial in cellular physiology because of the reactivity of its SH group [17]. Not only is the biosynthesis of Cys tightly controlled, but the degradation of this amino acid is also strong, leading to the inability of SeCys to continuously accumulate in large amounts intracellularly [10]. Therefore, it is important to investigate the SeCys degradation pathway.

Two types of enzymes that degrade Cys into sulfide, ammonia, and pyruvate have been identified: cysteine desulfhydrases [18] and cysteine desulfidases [19]. The former is a pyridoxal-5′-phosphate (PLP)-dependent enzyme, while the latter uses a [4Fe-4 S] center to catalyze the hydrolysis of cysteine to sulfide. Knockout of the *AecD*, cysteine desulfhydrase gene from *Corynebacterium glutamicum*, promotes Cys production [20]. Deletion of *TnaA* and *YhaM*, cysteine desulfhydrase and cysteine desulfidase respectively, from *Escherichia coli* [18, 21] significantly improved the Cys titer [21]. Another pathway for Cys degradation is catalyzed by cysteine desulfurase, which uses a pyridoxal phosphate center to mobilize sulfur derived from Cys to sulfur acceptor proteins and releases alanine [22]. In the case of bacteria, there are three distinct Fe-S cluster biosynthetic systems, the iron-sulfur cluster (ISC), sulfur mobilization (SUF), and nitrogen fixation (NIF) systems, and the corresponding cysteine desulfurases are IscS, SufS and NifS [23]. NifS and IscS are type I enzymes and SufS is a type II enzyme with a “shorter” catalytic loop for resistance to oxidative species [24] and iron starvation conditions [25]. NifS from *Arabidopsis thaliana* can also catalyze the conversion of SeCys to alanine and elemental Se [26]. IscS has dual functions in S and Se metabolism by synthesizing 5-methylaminomethyl-2-selenouridine [27]. Other pathways for the conversion of Cys are catalyzed by aminotransferases AspB and YhdR [28], Cys dioxygenase CdoA [29] and amino-acid racemase RacX [30] with 3-mercaptopyruvic acid, cysteine sulfinic acid and D-Cys as products, respectively. Nevertheless, the major routes for SeCys degradation in *B. subtilis* are unclear.

In the present study, three Mmts from marine bacteria were expressed separately in the SeMCys-producing strain GBACB to confirm the beneficial effect of MMet formation on SeMCys synthesis. Then, SeCys degradation pathways were analyzed via transcriptome analyses and candidate genes were knocked out. In addition, the serine (Ser) degradation pathway was weakened to promote SeCys formation. This study was the first to investigate the SeCys degredation pathway in *B. subtilis*, and the effect of bacteria derived Mmts on SeMCys production, providing insight into Se metabolism.

## Results

### The effect of MMet as a methyl donor on SeMCys production

**Fig. 2:**
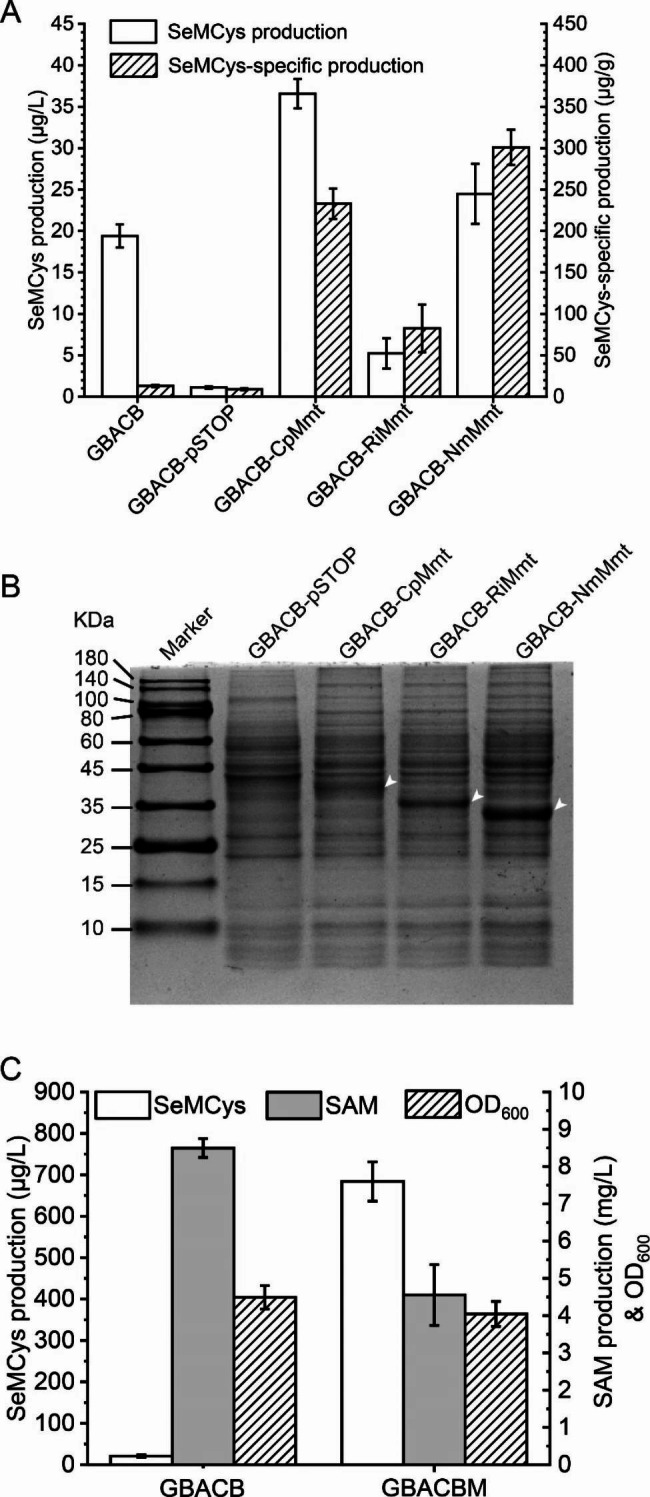
Screening exogenous Mmts for provision of MMet as a methyl donor for SeMCys production. **A**, Effect of expressing Mmts on SeMCys-specific production. All genes were codon-optimized based on *B. subtilis* and chemically synthesized. RiMmt was obtained from *Roseovarius indicus*, NmMmt from *Novosphingobium* sp. MBES04, and CpMmt from *Candidatus Peregrinibacteria*. **B**, SDS-PAGE analysis of the heterologous Mmts overexpressed in *B. subtilis*. White arrow shows the bends of target proteins. **C**, Influence of genome-integrated NmMMT gene on SeMCys production

SeMCys synthesis requires SMT to methylate SeCys, and both SAM and MMet act as methyl donors. In a previous study, enhanced SAM formation facilitated SeMCys production at 18 μg/L in *B. subtilis* GBACB [10]. In the present study, MMet was synthesized as a methylation donor. Three Mmts RiMmt, NmMmt and CpMmt from the marine heterotrophic bacteria *Roseovarius indicus* [15], *Novosphingobium* sp. MBES04 [16] and *Candidatus Peregrinibacteria* [15], respectively, were expressed in GBACB. As shown in Fig. 2A, the SeMCys-specific production of all Mmt-overexpressing strains increased compared to that of the untransformed strains, indicating that the synthesis of MMet greatly improved SeMCys formation. The vector pSTOP1622 dramatically inhibited biomass, and SeMCys production decreased significantly in the GBACB-pSTOP strain. Nevertheless, the SeMCys production in the CpMmt- and NmMmt-expressing strains was higher than that in the parental strain GBACB, and the NmMmt strain produced the highest SeMCys-specific production. Intracellular protein expression levels were shown in Fig. 2B, and each heterologous protein was distinguished by a distinct band.

*NmMmt* with a xylose-inducible promoter was integrated into the GBACB strain genome. NmMmt expression did not affect the biomass in either GBACB or GBACBM (Fig. 2C), as assessed by measuring the optical density at 600 nm (OD_600_). Extracellular SAM content decreased by approximately half. As SAM was the methyl donor for the synthesis of MMet from Met, the decline in SAM production indicated MMet formation in GBACBM, which resulted in an increase in SeMCys production from 20.7 to 687.4 μg/L.

### Prevention of SeCys degradation for enhancing SeMCys production

HTSATp, which is a SeCys overproducing strain, was compared with the bacterium HT using transcriptome analysis. Fragments per kilobase of exon model per million mapped reads (FPKM) was used to indicate the gene expression levels. Fold changes in gene expression related to Cys degradation were verified using real-time quantitative PCR (qPCR). Serine *O*-acetyltransferase (SATp and CysE) catalyzes Ser and acetyl-CoA into *O*-acetylserine, which was converted with selenide by *O*-acetylserine thiol lyase (CysK) to yield SeCys. The FPKM of SATp in HTSATp was 64,804, while that in HT was zero (Additional file 1: Fig. S1A), suggesting that SATp efficiently induced. Expression of SATp affected the following pathway and CysK transcription increased from 677 to 4680; the fold-change was confirmed by qPCR (Additional file 1: Fig. S1B). These results confirm a previous study that indicated *O*-acetylserine, the product of SATp, arrested the binding of the transcriptional regulator CymR and strengthened the transcription of *cysK* [17]. However, CysE expression levels were low, and the FPKM only changed from 34 to 78. Differential pathway enrichment showed that the Cys and Met metabolic pathways were most strongly influenced (Additional file 2: Fig. S2).

**Fig. 3:**
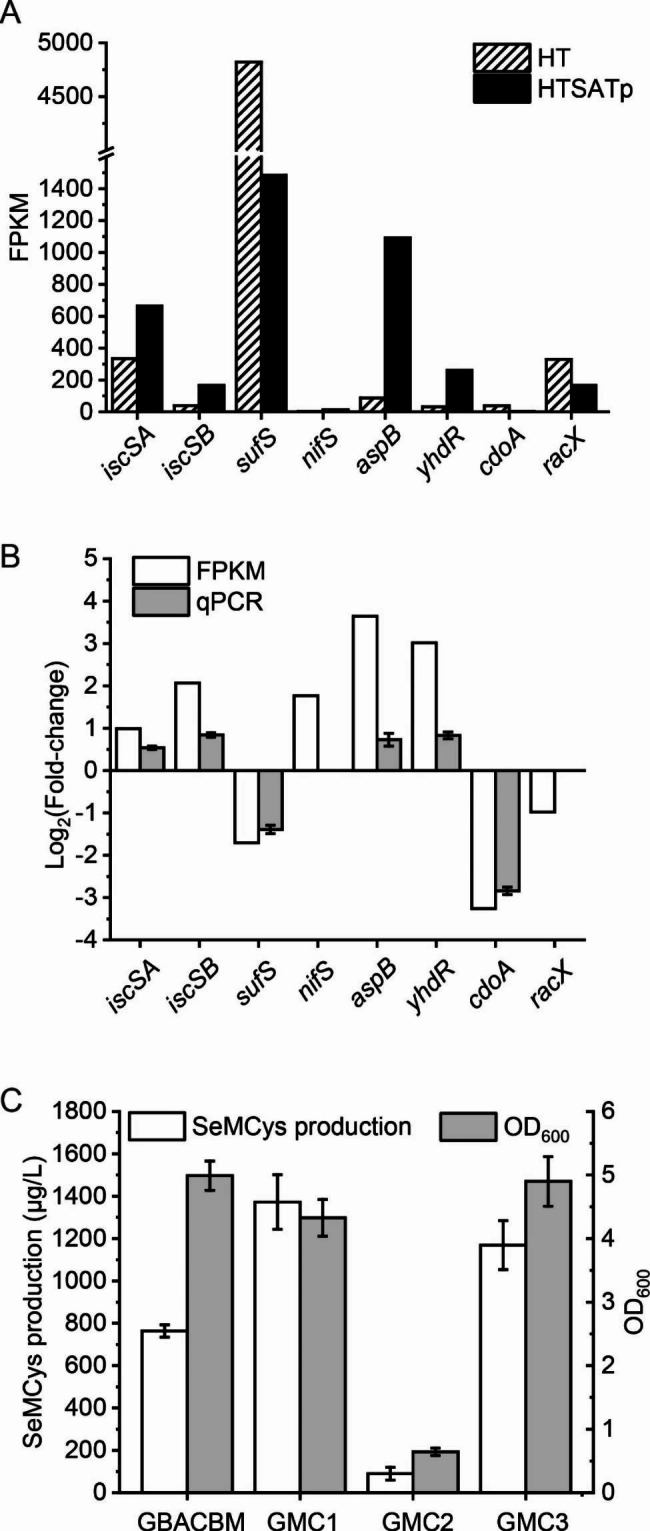
Inhibition of SeCys degradation pathway for enhancing SeMCys production. **A**, FPKM value of genes in the Cys degradation pathway. **B**, Fold-change of FPKM verified by qPCR. **C**, Influence of SeCys degradation gene deletion on SeMCys production

Neither enzyme families of cysteine desulfhydrases and cysteine desulfidases was found in *B. subtilis*. The transcription level of *sufS* in both strains was extremely high, but overproduction of SeCys decreased the FPKM value of *sufS* from 4822 to 1483 (Fig. 3A). The *cdoA* expression level was low and down-regulated under SeCys conditions, indicating a low metabolite flux toward Cys-sulfinate. RacX expression was down regulated, and RacX from *B. subtilis* preferentially racemizes arginine, lysine, and ornithine [30]; therefor, this pathway was not selected as a candidate for gene knockout. The upregulated genes at high SeCys levels were *iscSA*, *iscSB*, *nifS, aspB* and *yhdR* (Fig. 3A). IscSA and IscSB were used for the same reaction; therefore, only iscSB with a higher fold change in expression level was selected for gene knockout. Because the expression level of *nifS* was extremely low and could not be detected by qPCR (Fig. 3B), the gene was excluded as a candidate for deletion from the genome. Both AspB and YhdR were upregulated under high intracellular SeCys conditions. As these enzymes catalyze the transamination of amino acids to their corresponding α-keto acids [31], their broad substrate specificity made it necessary to check their effects on SeCys degradation. Finally, *iscSB, aspB* and *yhdR* were selected for deletion.

Interestingly, the disruption of *aspB* and *yhdR* had opposite effects on SeMCys production (Fig. 3C). Knockout of *yhdR* improved SeMCys production by 70.0%, but the lack of *aspB* dramatically decreased the SeMCys titer due to a decline in cell growth. Although AspB catalyzes 3-mercaptopyruvic acid formation [32], it may also play a more important role in other degradation pathways of amino acid metabolism [33]. In addition, *iscSB* knockout did not affect biomass and SeMCys production nearly doubled, indicating the inhibition of SeCys degradation pathway facilitated SeMCys synthesis.

### Inhibition of ser degradation pathway for enhancing SeMCys production

**Fig. 4:**
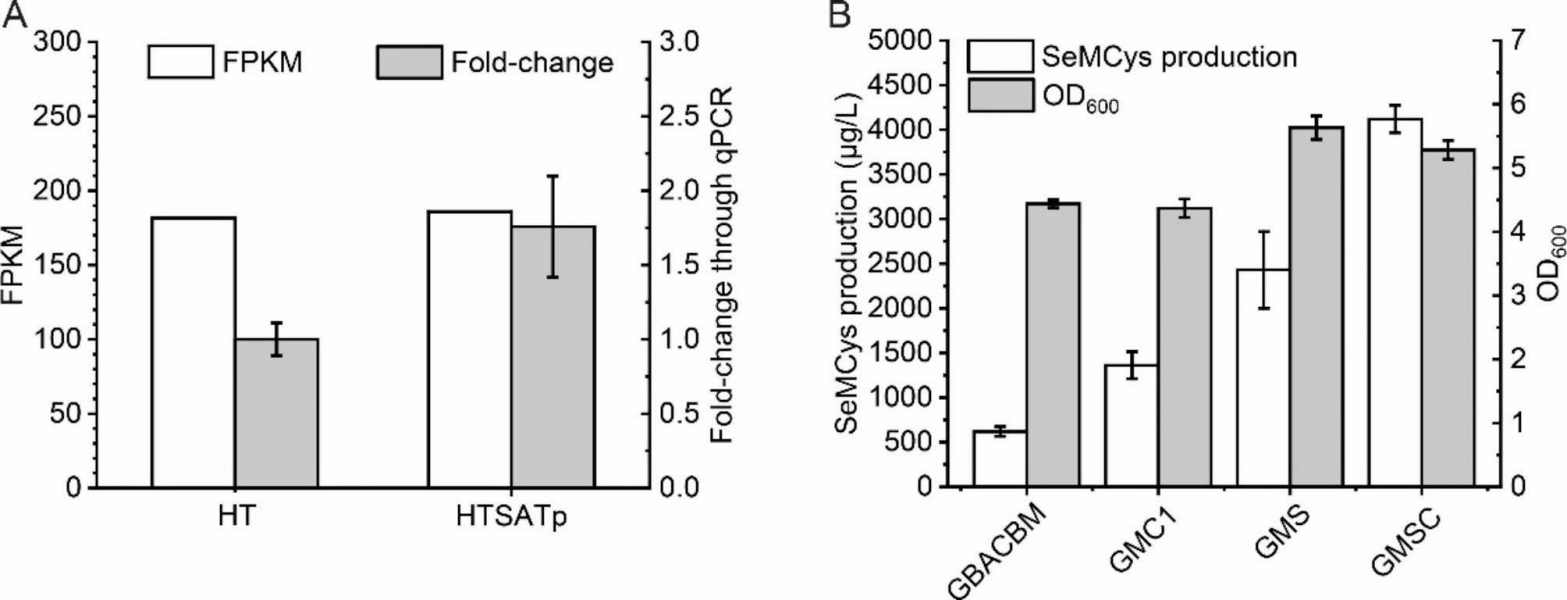
Knockout of *sdaA* for improving SeMCys production. **A**, Expression level of *sdaA*. **B**, Influence of deleting *sdaA* on SeMCys production

Ser is the precursor for the synthesis of both Cys and SeCys, and is beneficial for Cys production by preventing degradation reactions catalyzed by serine dehydratase encoded by *sdaA* [34]. Therefore, it was necessary to confirm the influence of *sdaA* on SeCys accumulation and SeMCys production. The *sdaA* expression level in HTSATp was almost the same as that in HT, according to FPKM, whereas qPCR showed a 0.76-fold increase (Fig. 4A). Knockout of *sdaA* in GBACBM induced the GMS strain, whose production of SeMCys increased 2.5 folds to 2430.1 μg/L. When both *sdaA* and *iscSB* were disrupted, the SeMCys titer increased to 4120.3 μg/L (Fig. 4B).

### Fermentation optimization for SeMCys production

Different media were used for SeMCys fermentation. Both Luria–Bertani broth (LB) and Terrific Broth (TB) were rich nutritional media, and there was no biomass difference between them. However, LB utilization produced higher levels of SeMCys with better specific production (Table 1). Synthetic culture medium (SM) provided minimal bacterial growth due to basic nutrition. Nevertheless, the maximum SeMCys-specific production was observed in SM. The medium contained a limited sulfur supply, which may have benefitted the metabolic fluxes to the Se pathway by sacrificing biomass. As the biomass in SM was extremely low, the formulation requires further optimization.

**Table 1:** Effects of different media on GMSC strain fermentation

medium	SeMCys production (μg/L)		SeMCys specific production (μg/mg DCW)	OD_600_
LB	4015.1 ± 183.6	2323.1 ± 63.7		4.94 ± 0.09
TB	1806.5 ± 4.4	1034.2 ± 30.5		4.99 ± 0.14
SM	443.5 ± 23.9	3454.8 ± 45.4		0.37 ± 0.02

**Fig. 5:**
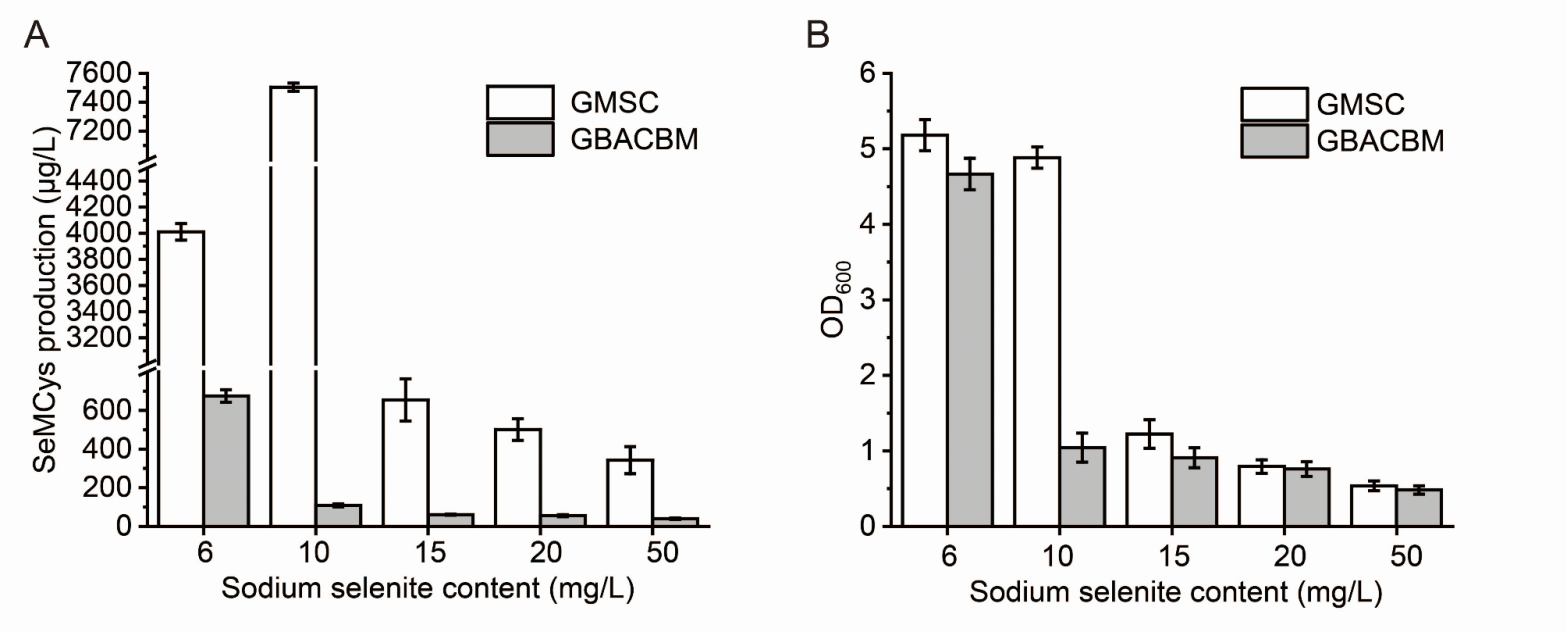
Optimization of sodium selenite content for GMSC fermentation compared to GBACBM. **A**, SeMCys production. **B**, OD_600_

The sodium selenite concentration was optimized for SeMCys fermentation in LB. For GBACBM, the optimal sodium selenite content was 6 mg/L, whereas that for the GMSC strain increased to 10 mg/L with a production of 7503 μg/L (Fig. 5A). The Se transformation yield was 71.3%. Furthermore, the GMSC strain was tolerant to higher sodium selenite concentrations (Fig. 5B) that concentration above 15 mg/L Na_2_SeO_3_ significantly affected cell growth, while that for GBACBM was only 10 mg/L.

## Discussion

SeMCys is synthesized via methylation of SeCys by SMT, which recognizes both SAM and MMet as methyl donor [11]. Because SAM is also a precursor of MMet [35], a SAM-producing strain can be used to synthesize MMet if Mmt is introduced into the strain. In this study, the RiMmt, MmMmt and CpMmt genes were codon-optimized based on *B. subtilis*, and the *NmMmt*-transformed strain that expressed the highest heterologous protein level exhibited the maximum SeMCys production (Fig. 2A). In addition, NmMmt overexpression consumed SAM to produce MMet, which facilitated SeMCys formation (Fig. 2C).

However, an attempt to introduce MMT from *(A) thaliana* into *S. cerevisiae* SAM-producing strains harboring the SMT gene had no beneficial effect on SeMCys production [9]. This may be because MMT also catalyzes the synthesis of *Se*-methylselenomethionine by methylating selenomethionine (SeMet) [36], which is the main selenoamino acid in Se-enriched yeast [37] and is simultaneously the major precursor for the synthesis of SeCys in *S. cerevisiae* [9]. Unfortunately, Se-methylselenomethionine is also a precursor of volatile Se [38], leading to a loss of absorbed Se. Overexpression of MMT originating from *Arabidopsis* in *E. coli* produced 10 times more volatile Se than the parental strain when both strains were supplied with SeMet [36]. However, Mmt substrate specificity requires further investigation. In addition, the *(B) subtilis* strain GBACBM did not accumulate SeMet intracellularly (data not shown), preventing the formation of *Se*-methylselenomethionine and further volatile Se, and the efficiently formed MMet promoted SeMCys production. Although the Met-to-Cys conversion pathway also exists in *B. subtilis* (Fig. 1) and is under the control of MccA and MccB [39], the absence of both cytoplasmic and extracellular SeMet in GBACBM strain indicates that SeMet contributes little to SeCys formation. In summary, low SeMet levels may be required for efficient SeMCys production.

The major routes of SeCys degradation were also explored. High intracellular SeCys levels led to the upregulation of i*scSA*, *iscSB* and *nifS* at the transcriptional level (Fig. 3A and B), and iscSB knockout significantly improved SeMCys production (Fig. 3C), indicating that degradation of SeCys to Ala was an important route to decrease intracellular SeCys. In addition, *yhdR* and *aspB* were found at higher transcription levels during SeCys accumulation, and the deletion of *yhdR* promoted SeMCys production, indicating that 3-mercaptopyruvate is another SeCys degradation route. To the best of our knowledge, this is the first study to explore the SeCys degradation pathway in *B. subtilis*. However, as Cys is another substrate of IscSB [31], and both YhdR and AspB catalyze acidic and neutral amino acids [40], further studies are needed to determine the substrate specificity of each enzyme.

Ser is the precursor of both Cys and SeCys, and the overexpression of Cys insensitive SATp successfully improves SeCys production [10]. Based on the improvement in the expression levels of pathway enzymes, further knockout of *sdaA* may promote intracellular content of Ser, which is a substrate of SATp; the strain promoted SeMCys production by 2.5-fold. In addition, the biomass of both *sdaA* deletion strains (GMS and GMSC) increased. Ser is the precursor for *O*-acetylserine, which is further catalyzed to synthesize SeCys by fixing selenide. As selenide is toxic to cells due to the formation of superoxide [41], acceleration of SeCys synthesis by enhancing Ser level may consume selenide, reducing the toxicity of selenide and increase biomass.

The metabolic network inside the cell was complex. Se competed with sulfur as substrate of many enzymes, but methyl donors for SMT came from sulfur-containing amino acids. Therefore, the precise regulation of metabolism needs further study to achieve the highest SeMCys production. It needs to use synthetic culture medium to carry out the precise control of sulfur, and it also needs study on metabolomics analysis to reveal the metabolic balance of sulfur and Se in high-yield SeMCys strains.

SeMCys can be naturally synthesized in Se-hyperaccumulating plants, especially *Astragalus* species [42]. *(A) bisulcatus* accumulates SeMCys as a major seleno-compound at a content of 52.2 μg/g fresh weight in the shoots [43]. In microbes, only *S. cerevisiae* and *(B) subtilis* have been used as hosts for metabolic engineering to produce SeMCys. The highest SeMCys production in selenized *S. cerevisiae* was only 1.140 μg/g DCW [9]. In our previous work, *B. subtilis* was engineered to produce SeMCys for the first time. Through overexpressing SMT and enhancing synthesis of SeCys and SAM as methyl donor, the final strain GBACB showed an extracellular production of SeMCys at 18.4 μg/L [10]. The present work helped increase SeMCys production up to 300-fold on compared to that seen in the previous report, making the production capable of achieving mg/L yields.

## Conclusions

In this study, we demonstrated that MMet, as a methyl donor, was more conducive to the synthesis of SeMCys than SAM in vivo. The main SeCys degradation pathways in *B. subtilis* were revealed for the first time, and inhibition of both SeCys and Ser degradation pathways benefited SeMCys production. The flask fermentation of GMSC represented the highest reported SeMCys yield to date for any organism. These results can be used to guide commercial practices for SeMCys biosynthesis, and will facilitate the formulation of new Se dietary supplements.

## Materials and methods

### Strains and reagents

The strains and plasmids used in this study were listed in Table 2. The *Escherichia coli* DH5α was used for cloning and vector construction. The *B. subtilis* GBACB constructed in our laboratory was used as the initial strain for genomic manipulations. Plasmid pSTOP1622 was used as a vector for gene expression, and plasmids pHT-XCR6 and pcrF11 were used for gene editing.

**Table 2:** Strains used in this study

Strains/plasmids	Genotype	Source
Strains		
*E. coli DH5α*	*F*−, *endA1*, *hsdR17* (*rk*-*mk*-), *supE44*, *thi*1, *recA*1, *gyrA*, (Nalr), *relA*1, D (*lacZYAargF*), U169, and F 80*lac*ZDM15	Lab stock
*B. subtilis* 168		Lab stock
GBACB	*B. subtilis* 168; Δ*amyE*::P_*grac*_-*SATp*-P_*43*_-*SMT*; Δ*xylA*::P_*grac*_-*SAM2*-*SMT*	[10]
GBACB-pSTOP	*B. subtilis* GBACB; pSTOP1622	This work
GBACB-NmMmt	*B. subtilis* GBACB; pSTOP1622- NmMmt	This work
GBACB-RiMmt	*B. subtilis* GBACB; pSTOP1622- RiMmt	This work
GBACB-CpMmt	*B. subtilis* GBACB; pSTOP1622- CpMmt	This work
GBACBM	*B. subtilis* GBACB; Δ*KinB*::P_*xylA*_-*NmMmt*	This work
HT	*B. subtilis* 168; pHT01	[10]
HTSATp	*B. subtilis* 168; pHT-SATp	[10]
GMC1	*B. subtilis* GBACB; Δ*KinB*::P_*xylA*_-*NmMmt* ; Δ*iscSB*	This work
GMC2	*B. subtilis* GBACB; Δ*KinB*::P_*xylA*_-*NmMmt* ; Δ*aspB*	This work
GMC3	*B. subtilis* GBACB; Δ*KinB*::P_*xylA*_-*NmMmt* ; Δ*yhdR*	This work
GMS	*B. subtilis* GBACB; Δ*KinB*::P_*xylA*_-*NmMmt* ; Δ*sdaA*	This work
GMSC	*B. subtilis* GBACB; Δ*KinB*::P_*xylA*_-*NmMmt* ; Δ*iscSB* ; Δ*sdaA*	This work
Plasmids		
pSTOP1622	Amp^r^, Tet^r^, *E. coli*-*B. subtilis* shuttle vector	[49]
pSTOP1622- NmMmtN	pSTOP1622 derivate with *NmMmt* cloned	This work
pSTOP1622- RiMmtN	pSTOP1622 derivate with *RiMmt* cloned	This work
pSTOP1622- CpMmtN	pSTOP1622 derivate with *CpMmt* cloned	This work
pHT-XCR6	pHT01 derivate, XylR-P_*xylA*_-FnCpf1-NgAgo (D663A, D738A, truncated to 650–887 aa)	[44]
pcrF11	ColE1 Kan^r^, RepF Kan^r^, *E. coli*-*B. subtilis* shuttle vector, crRNA insertion under P_*veg*_	[44]

All the chemicals were purchased from Sangon Biotech (Shanghai, China). Plasmid extraction, DNA gel purification and RNA extraction kits were purchased from TIANGEN Biotech (Beijing, China). Restriction enzymes, T4 DNA ligase, PrimeSTAR HS DNA polymerase, SYBR Premix Ex Taq and PrimeScript RT reagent kit with gDNA eraser were purchased from Takara Biomedical Technology (Beijing, China). Taq DNA polymerase used for colony polymerase chain reaction (PCR) was purchased from Zoman Biotechnology (Beijing, China). Seamless cloning and BCA protein assay kits were purchased from Beyotime Biotechnology (Shanghai, China). Oligonucleotides were synthesized by GENEWIZ (Suzhou, China).

### Genome manipulation and plasmids construction

Genome manipulation was performed using CRISPR/Cpf1 system and described in the previous study. pSTOP1622 was used for gene expression analysis. The primers used in this study were listed in Additional file 3: Table S1. The heterologous gene sequences of *RiMmt*, *NmMmt*, *CpMmt* were optimized for expression in *B. subtilis* (Additional file 4: Table S2) and synthesized by GENEWIZ (Suzhou, China). CRISPR associated proteins systems was used for genome integration and gene knockout [44]. The detailed procedures of recombinant plasmid construction are described in the Supporting Information.

### Cultivation conditions and sample preparation

LB medium (10 g/L tryptone, 5.0 g/L yeast extract, and 10 g/L NaCl) was used as the fermentation medium. Isopropyl-β-D-thiogalactoside (IPTG) at a final concentration of 1 mmol/L or 5 g/L xylose was added to the medium to induce protein expression under the control of the P_*grac*_ or P_*xylA*_ promoter, respectively. Sodium selenite at 6 mg/L was added when OD_600_ reached 1.0. For shake-flask fermentation experiments, three biological transformants of each strain were selected and cultured at 33 °C with 220 rpm agitation for 12 h in 250-mL flasks containing 40 mL LB broth. The samples were centrifuged at 10,000 rpm at 4 °C for 1 min. The pellet was washed twice with ultrapure water and frozen in liquid nitrogen. Both the pellet and supernatant were stored at -80 °C until further use.

TB (20 g/L tryptone, 24 g/L yeast extract, 72 mM K_2_HPO_4_, 17 mM KH_2_PO_4_, 4.0 g/L glycerol) and synthetic medium (SM), which contained 2% glycerol, 1 mM (NH_4_)_2_SO_4_, 150 mM NH_4_Cl, 5 mM potassium phosphate, 4 mM trisodium citrate, 2 mM MgCl_2_, 0.7 mM CaCl_2_, 50 μM MnCl_2_, 5 μM FeCl_3_, 1 μM ZnCl_2_, 2 μM CuCl_2_, 3 μM CoCl_2_, 2.5 μM Na_2_MoO_4_ and 0.25 mM of L-tryptophan, pH 7.0, were also used for SeMCys fermentation.

### Determination of selenoamino acids and SAM concentration through ultra-performance liquid chromatography-tandem mass spectroscopy (UPLC-MS/MS)

The Acquity XEVO TQ UPLC system (Waters, Milford, USA) was used for seleno-amino acid determination [45] and SAM determination [46], with slight modifications [10]. Briefly, chromatography was performed on a Waters Acquity UPLC HSS T3 C18 column (2.1 mm × 100 mm, 1.8 μm particle size) using 0.1% formic acid aqueous solution and acetonitrile to form a gradient mobile phase. TQ mass spectrometer was set in the positive ESI mode. The precursor-product ion transitions of *m/z* 198.0 → 180.9 was for SeMet, 337 → 247.8 for selenocystine (SeCys_2_), 184.0 → 166.89 for SeMCys, and 399 → 250 for SAM. The optimal MS detection conditions were as follows: capillary voltage, 2.17 kV; source temperature, 150 °C; desolvation temperature, 500 °C. The cone voltage was set to 22 V for SeMet, SeMCys, and SAM, and 30 V for SeCys_2_. The collision voltages of SeMet, SeCys_2_, SeMCys and SAM were 8, 13, 15, and 12 eV, respectively.

### Transcriptome analysis

For batch cultivation in a 1-L bioreactor (Minifors, INFORS HT, Basel, Switzerland), strains were grown at 33 °C and 800 rpm at an aeration rate of 1.0 vvm in a total culture volume of 0.7 L. IPTG was added to the fermentation medium at 1.5 h and sodium selenite at 6 mg/L was added after inoculation for 2.5 h. Samples were taken at 4 h for HT and 5.5 h for HTSATp. Samples for RNA-sequencing were centrifuged for 1 min at 4^o^C and 10,000 rpm to remove the supernatant, the pellets were frozen in liquid nitrogen and sent to Allwegene (Beijing, China) for RNA-sequencing.

RNA was extracted using the RNAprep Pure Cell/Bacteria Kit (TIANGEN, Beijing, China). RNA integrity was confirmed using an Agilent 2100 Bioanalyzer (Agilent Technologies, Santa Clara, CA, US). Qualified total RNA was further purified using RNeasy Micro Kit and RNase-Free DNase Set (QIAGEN, Hilde, Germany). Purified total RNA was digested to eliminate rRNA. The RNA was then fragmented by heating at 94°C and used to synthesize single-strand cDNA with random hexamers: it was then synthesized into double-strand cDNA. After purifying with AMPure XP beads, the double-stranded cDNA was adenylated at the 3’ end, then ligated to the sequencing adapters. Pair-end sequencing samples were selected according to the length of the fragment and amplified through PCR to construct a cDNA library. Finally, the libraries were sequenced on Illumina HiseqTM2500/4000 (Illumina, San Diego, CA, USA) at Beijing Allwegene Technology Co., Ltd (Beijing, China).

Clean reads were aligned to the *B. subtilis* 168 genome (Genbank accession number NC_000964.3) using Bowtie2. The raw data produced using RNA-sequencing were deposited in the National Center for Biotechnology Information database under the accession number PRJNA785290 (https://www.ncbi.nlm.nih.gov/bioproject/PRJNA785290). Each sample was analyzed for gene expression levels using HTSeq software using the Union model [47]. Gene expression levels in each library were normalized to FPKM. FPKM value of 1 was used as the threshold for judging whether or not the gene was expressed, and only the genes with FPKM > 1, were analyzed. Differentially expressed genes between two samples were identified with a log2 foldchange of > 1 and a q-value of < 0.005 as the threshold.

### qPCR

For verifying transcriptome data, qPCR was performed by LightCycler 480 system (Roche, Germany). The primers were designed by Beacon Designer 8 and the sequences were listed in Additional file 2: Table S1. The genes’ expression levels of HTSATp and HT were compared. The fold change was calculated using the 2^−ΔΔCt^ method [48]. Differentially expressed genes between two samples were identified with a log2 foldchange of > 1 and a q-value of < 0.005 as the threshold.

### Electronic supplementary material

Below is the link to the electronic supplementary material.

Supplementary Material 1

## Data Availability

The data used and /or analysed during the current study are available from the corresponding author on reasonable requests.
