# Supplementary material for: Optimized methyl donor and reduced precursor degradation pathway for seleno-methylselenocysteine production in Bacillus subtilis
Source: Microb Cell Fact. 2023 Oct 19;22:215. doi: 10.1186/s12934-023-02203-1 (PMC10585787; doi:10.1186/s12934-023-02203-1)
Supplement: Supplementary file 1 — Supplementary Material 1 [file 12934_2023_2203_MOESM1_ESM.docx]

Microbial Cell Factories

**Supporting Information**

Optimized methyl donor and reduced precursor degradation pathway for seleno-methylselenocysteine production in *Bacillus subtilis*

**Xian Yin^1,2^, Meiyi Zhao^1,2^, Yu Zhou^1,2^, Hulin Yang^1,2^, Yonghong Liao^1,2*^, Fenghuan Wang^1,2*^**

^1^Beijing Advanced Innovation Center for Food Nutrition and Human Health, Beijing Technology and Business University, Fucheng RD 11, Beijing 100048, China

^2^School of Light Industry, Beijing Technology and Business University, Fucheng RD 11, Beijing 100048, China

***Corresponding author:**

liaoyh@th.btbu.edu.cn, phone: +86 1068988710

wangfenghuan@th.btbu.edu.cn, phone: +86 1068985252


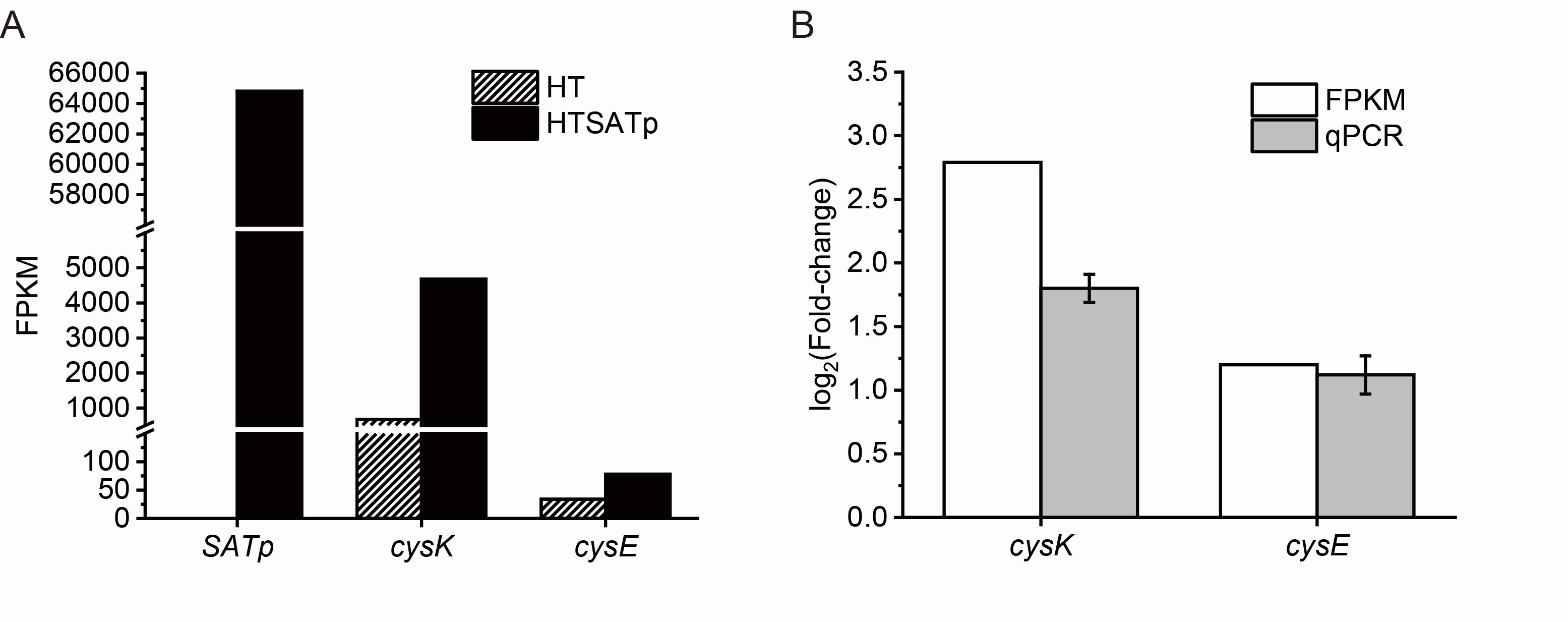
**Additional file 1: Fig. S1.** Expression level of genes in Cys synthesis pathway. A, FPKM value. B, Fold-change of FPKM that verified through qPCR.


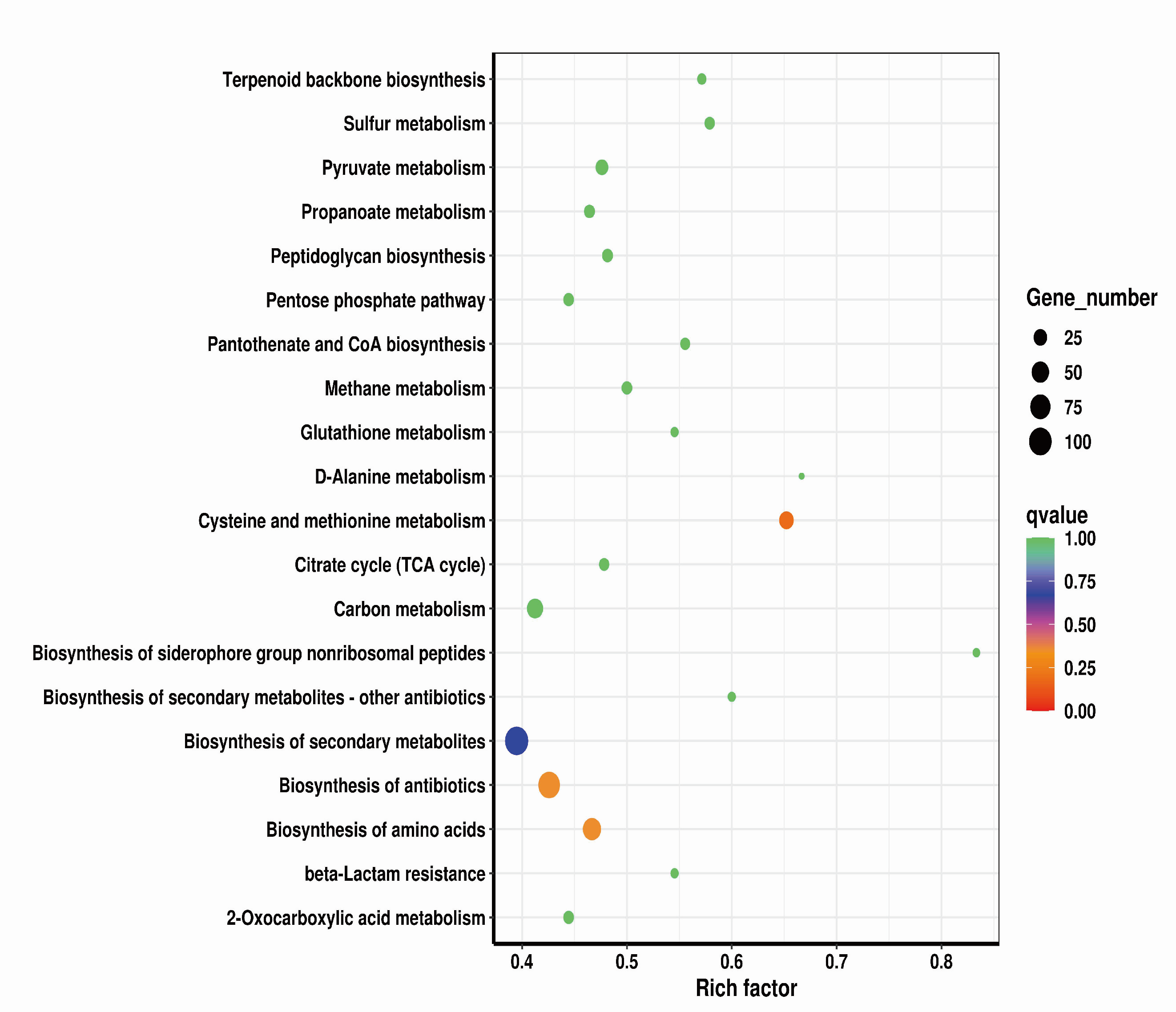


**Additional file 2: Fig. S2.** Differential pathway enrichment (HTSATp vs HT).

**Additional file 3: Table S1.** Primers used in this study.

| Primer | Sequence |
| --- | --- |
| kinB-up-F | CAATCGATTTTGAAGATTTG |
| kinB-up-R | CGTATAAAATATGAATCTATTATAACAC |
| kinB-down-F | CGCGCGGGTACCCGCTGATCTTCCTCACTAGCAA |
| kinB-down-R | CGCGCGGAATTCACGAAGGAATGAGATTCGAGGA |
| mmt-kinB-F | GTGTTATAATAGATTCATATTTTATACGATCTCTAGAGCCGTCTGTACGTTCCTTAAGA |
| mmt-kinB-R | TTGCTAGTGAGGAAGATCAGCGGGTACCGGGTTAATCCATTTTCGGTGTT |
| kinB-444-F | AGATCGCCTCAAGCTGGATTCTTT |
| kinB-444-R | AATTAAAGAATCCAGCTTGAGGCG |
| Mmt-F | TTCGAGGATTTCATCCGTAAAC |
| Mmt-R | GATTTGGAGACAAAGCTTAGTGA |
| iscSB-L-F | ATTTGTGCTTGTTGCACCTAAAT |
| iscSB-L-R | GTAAGGCTTCGTCGTTGAACTAT |
| iscSB-R-F | CGCGGTACCAATTAAGGGAAATGATGAGGTAGA |
| iscSB-R-R | CGCGAATTCGATGATTTCTGTTTTGTCCATG |
| aspB-L-F | TTGCAGGGGAAAAAGAATAA |
| aspB-L-R | GGTGTTAATGCGGATACTCT |
| aspB-R-F | CGCCTGCAGTGAAAGAATCAAGCGTTTTG |
| aspB-R-R | CGCAAGCTTGCTCAAGTTTTGACGTGTCT |
| yhdR-L-F | TGATTCACCTAAAACCCCTAATC |
| yhdR-L-R | AAACAGCTTGCGAATCCATG |
| yhdR-R-F | CGCGGATCCGATTAAAAACTCACGGGACATT |
| yhdR-R-R | CGCGGTACCAACGGAAACAGGAGCAAATA |
| sda-L-F | CCGGGAAACGTGTTAAACTA |
| sda-L-R | AACGCTTCTGTATTTCATGCTA |
| sda-R-F | CGCGGTACCGGTTCACGTGAAACAACATCAG |
| sda-R-R | CGCGAATTCGGGAGGCTTTTGATAAATCT |
| iscSB-156-F | AGATTTAGGCCTAAGGACCTTTTG |
| iscSB-156-R | AATTCAAAAGGTCCTTAGGCCTAA |
| aspB-198-F | AGATTCAGTTCAGCCAAACCACCG |
| aspB-198-R | AATTCGGTGGTTTGGCTGAACTGA |
| yhdR-129-F | AGATACTCGGCAACCCGATTGTTG |
| yhdR-129-R | AATTCAACAATCGGGTTGCCGAGT |
| sda-371-F | AGATAATCTTTCCTCCGCCAATCG |
| sda-371-R | AATTCGATTGGCGGAGGAAAGATT |
| gapA-real-F | TGCTGAACTGAACCAAGA |
| gapA-real-R | TTGTGTAAGAGTGAACAGTTG |
| iscSA-qPCR-F | AGACAGAAGGTCAATATG |
| iscSA-qPCR-R | AATAATGAAGTCGGAACA |
| iscSB-qPCR-F | AAACAGGCAAACATATCATT |
| iscSB-qPCR-R | AATCCGTCTTCATTCACA |
| sufS-qPCR-F | ATACACTTGGAACCAGAG |
| sufS-qPCR-R | TTAACGCCACCATATTCA |
| nifS-qPCR-R | GAGGCATTAAGAACATAC |
| nifS-qPCR-F | CATAACATATTGTCCTTCA |
| aspB-qPCR-F | GAAAGCCTGTTTATGTAG |
| aspB-qPCR-R | ATAGTTCTTCTTCCGTATA |
| yhdR-qPCR-F | TTCTCAATACACCGCATA |
| yhdR-qPCR-R | CATCAAACAGCACATAAATC |
| cdoA-qPCR-F | ATTATCGTGATTAACATTCC |
| cdoA-qPCR-R | AAGTATGAATTGGAGAGTT |
| racX-qPCR-F | ATTGTCAGTTCCTATGTT |
| racX-qPCR-R | GAATAACTTCTTGTCCATT |
| sdaA-qPCR-F | ATTACAAGCCGTATTCCA |
| sdaA-qPCR-R | TCTCTAATTCTCTTCCTGTC |
| cysK-qPCR-F | TTAACAACCCTTCTAACC |
| cysK-qPCR-R | GTTCAACAGCATAAATCTT |
| cysE-qPCR-F | TATCTCAAGTAAGCCGATT |
| cysE-qPCR-R | TTATTGCCGATTTCACAT |

**Additional file 4: Table S2.** DNA sequences of heterologous genes used in this study.

| Name | Sequence | Ref. |
| --- | --- | --- |
| *RiMmt* | ATGGGAGATAGCGAAGAACCGGTTGTCACACCTCATGCACCGGAATTTGCATTTGATCCGACAGATCCGTGGACAGAAACGTTTCAACGCGGACTGGAAATTGCAGGACTTGGAGGCAAAAGAGTCTACGAAGTTGGCATTGGCACAGGAATCAACGTCGCGTTCATGCTGCAGATTTGCGAAGCAGCACTGGTTTCAGGAAGCGATCTTGATCCTAGATTAGCAGGCCTGGCAGAAAGAAATGTCAGAGATCTGGCACCGAGAAGAGCAGATAGATTTCATCCGGTCGAAGGCGCAGTTTCACTGATCGATACACCGGAAGCAAGAGCACAAGTTGGCAGATCAGACGTCATTGTTGGCTGTCTTCCGCAAGTTGGAGAACCAGACGATGTTAGACTGAGAGCGTTTCGCACAGCACAAAAAGCGAAACTGGCGAAAGGCGCAGATACACGCGACGAAGATCATATCGCGCATTACTACCCTTGGGCGGAATTTGATAGCTACCCGTTTAATAGCGTTGGACTTGGCCTGAACGAAGCACTTCTGAGAAGAACAAGAGCAACAGCACCGGCAGCAGACGTTGTTCTGAATTTTGGCGCAAGAGTTGGCTCAGCAGTCCTGTTTGAACTGTTCGAAGCGAACGGATACGTCCCGGAAAAACTGCATAGCCAAATTGTCCTGCAACACGCAGGCACAGATATTAGCTTCTTTGTCGCGCTGGAAAATGCGCTTGCACAAACAGGCCTGGAACGCGAATTCACTTGCGAATTCTACGGCGATCCGGAAGGAGCAACAAGACTTTCAGCAACAGAAGCGCAAGCGCTTGTTGATACAGATAGCGCGGCGGAAATCTATCACGAAGTTTGCGTTATTAGAGGCAGACCGGCACTTAGCGAAAACGATCCGAGCGATTCATAA | [1] |
| *NmMmt* | ATGTCAGACGCAGACGGCAGCAAAGTCATTTCAAGACACGACGATCCGTCAGCAACAACAGAAAGACCGGGATACGCATTTGATCCGACAGATCCGTGGACAATCACGTTTCAACAAGGCCTGAAAGCAGCAGGACTGGAAGGAAAAGCAGTGTATGAAGTTGGCGTTGGCACAGGAACAAATGTCGCGTTTGTCCTGAGACATTGCGCAGCGAAAGTCTTTTACGGCAGCGATCTTGATCCGAGACTGGTTGAACTGGCAAGAAGAAACGTCGCAAATCTTGCACCGGAAAGAGCAGATAGCTTTCAACCGGTCGAAGGAGCAGTTTCACTGATCGATACAGACGAAGCGAGAGCGAAAATTGCGAGAACAGACGTCGTCATTGGTTGCTTACCGCAAGTTGGAGATCCGAACGACGAAAGATTTGCAGCATTTCGCGCAGAACACGCAGTCGATTTACCTCAAGGAGCAGACGACGAAGCGCAAGATCATATCGCGCATTACTATCCGTGGGCAATGTTCGACGAGTATCCGTACAACTCAGTTGGCCTTGGACTGAACGAAGCACTTCTGAGACGCATCAAAGAACAGGCACCGAAAGCGGATGTCGTCATGAACTTTGGTTGCAGAATCGGCAGCGATCTGATCTTCGAGATGTTCAGAGCAAACGGCTACGAACCGGAAAAACTGGCATCACAACTGGTTCTTCAACACGCAGGAACGGATATCAGCTTCTTCGTCACGCTGGAAGGAGCATTAACAGGAACAGATCTGGAAGGCGAATTCGTTTGCCGCTTTTTCGCAGATCCGCTTGGACACGAACCGTTATCAGCAAGAGCAGCACAAGCACTGCTGGATAAAGATCCGAACGTCCCGCTGTATCACGAAGTTGCAGTCATTAGAGGAACACCGAAAATGGATTAA | [2] |
| *CpMmt* | ATGAGCATTAGCACGCCTTGCAGCAGAGAAGATCTGATTGTCCCGCAGTACGCGTTTGTTCCGGACGCTTGGACAACAACATTTCAGGAAGGCCTGCTGGCAGATAAATCAGAAATCGTCGGCAAAAGACTGCTGGAAGTTGGAGTTGGAACAGGAGCAATGGCGATTTTTGGCCTGACAAAACTGGAGGTGGCACATTATTACGGCAGCGATCTTGATCCGAATTTACCGACACTGGCAAGCCAGAACATCGAAAGAATTTGCCCGGACGAAGCATCACGCTTTATGCCGATTCTGGGAAGCACAAATCTGCTGACACCGGTCGATAAGAACAATCGCAGCATCATCCTGGAGACGGATATCGTCATCGCTTGCATTCCGCAGGCAATTAGACCGCCGGATAAAGAAGTTTGCTTCGACGATCTGGCGCATTATTATCCGGGAAACCTGTTCGAGAACTACCAGTTCAACAGCCTGGCACTTGGACTGAACGAAGCACTGCTGGAACAATTTATGAGCGTCGTCCGCGAAACGAAAGACTACAACAAGCGCATCTACCTGAACATTGCGGGCAGAGTCGGCCTGGAAAACATCAAAGAACTGTTTAAGAGCCACGGACTGACAAGCGAAATTGTCCATCAGGTCGTCATTCCTCAGTGCCCGTCAACAAGCCTGCAGTTTTTCGTTGACATCGAAACAGCGAGCACAGATAACGCGTTCAAGTGCGAATTCTATAGCGATCCGGAAGGCAATTGCCAGATCAGCGCAAACGAAGCGGAAAAACGTCGCCTGCAGAAAGATCCGGTCTTTCACAAGCTGTACGTCATCAAAGGCATTCCGCAGCTGAATAGCGTCAACCTGAACCAGTTCCTGATCACGTTCCTGGATTGCATTCGCCAACAACTGGCGCAGCTGTACAAAAATCGCGAAAGCAGCCCGCTGCTTCCGTATTATAACTAA | [1] |

**Plasmid and strain construction**

The heterologous genes of RiMmt from *R. indicus*, NmMmt from *Novosphingobium sp.* MBES04 and CpMmt from *C. Peregrinibacteria* were optimized for expression in *B. subtilis* and synthesized, then ligated into pSTOP1622 digested by *Spe* I and *Bam*H I with T4 DNA ligase to construct plasmids pSTOP1622-*RiMmt*, pSTOP1622-*NmMmt* and pSTOP1622-*CpMmt*, respectively.

For the integration of P*_xylA_*-*NmMmt* into *KinB* site of the *B. subtilis* 168 genome, donor DNA was constructed. The upstream region was amplified with kinB-up-F/kinB-up-R, using the genome as a template and connected to pMD19 plasmid (Takara Biomedical Technology, Beijing, China). The downstream region was amplified with kinB-down-F/kinB-down-R, digested with *Kpn* Ⅰ and *EcoR* Ⅰ, and connected to pMD19 with upstream region, which was already digested with *Kpn* Ⅰ and *EcoR* Ⅰ. Subsequently, P*_xylA_*-*NmMm* fragment was amplified with mmt-kinB-F/mmt-kinB-R using pSTOP1622-*NmMmt* as the template, and connected to pMD19 with both upstream and downstream region using a seamless cloning kit to form pMD-LB-Mmt-RB. The primer pair Mmt-F/Mmt-R was used to amplify the donor DNA of LB-Mmt-RB for integration using pMD-LB-Mmt-RB as a template.

The CRISPR RNA (crRNA) used in this study was designed using the tool called CRISPR-DT [3]. crRNA construction method using the synthetic oligo mediated assembly of the crRNA array has been described by Wu et al. (2020) [4]. A pair of oligonucleotides, kinB-444-F/kinB-444-R, was synthesized and annealed to form a double strand targeting sequence with cohesive ends. The annealing product was then diluted to 1 μmol/L and ligated between two direct repeats of plasmid pcrF11 digested with *Eco* 31I.

To construct the GBACBM strain, pHT-XCR6 was transformed into the parental strain GBACB. The crRNA in pcrF11 and the donor DNA, LB-Mmt-RB, were then co-transformed. The transformants were screened on LB agar plates containing chloramphenicol, and the genotypes were confirmed by PCR. Finally, the strain was cultured in LB broth with 0.005% SDS overnight in a shake flask to eliminate pHT-XCR6 and pcrF11, and chloromycetin and kanamycin resistance on the plate was confirmed.

To construct the strains of GMC1, GMC2, GMC3, GDS and GDSC, all steps were the same, except for the change of primers to construct the donor DNA and pcrF11 derivatives.

**References**

1. Peng M, Li CY, Chen XL, Williams BT, Li K, Gao YN, Wang P, Wang N, Gao C, Zhang S, et al. Insights into methionine *S*-methylation in diverse organisms. Nat Commun. 2022;13(1)**:**2947. https:// doi. org/ 10.1038/ s41467-022-30491-5.

2. Williams BT, Cowles K, Bermejo Martinez A, Curson ARJ, Zheng Y, Liu J, Newton-Payne S, Hind AJ, Li CY, Rivera PPL, et al. Bacteria are important dimethylsulfoniopropionate producers in coastal sediments. Nat Microbiol. 2019;4(11)**:**1815-25. https:// doi. org/ 10.1038/ s41564-019-0527-1.

3. Zhu H, Liang C. CRISPR-DT: Designing gRNAs for the CRISPR-Cpf1 system with improved target efficiency and specificity. Bioinformatics. 2019;35(16)**:**2783-9. https:// doi. org/ 10.1093/ bioinformatics/ bty1061.

4. Wu Y, Liu Y, Lv X, Li J, Du G, Liu L. CAMERS-B: CRISPR/Cpf1 assisted multiple-genes editing and regulation system for *Bacillus subtilis*. Biotechnol Bioeng. 2020;117(6)**:**1817-25. https:// doi. org/ 10.1002/ bit.27322.
